# The relationship between emotional dysregulation and, HIV acquisition risk behaviours and intimate partner violence perpetration among young men in rural areas and urban informal settlements in South Africa

**DOI:** 10.1080/13548506.2026.2635751

**Published:** 2026-03-02

**Authors:** Princess Nyoni, Andrew Gibbs, Smanga Mkhwanazi, Andrew Tomita

**Affiliations:** a School of Nursing and Public Health, Discipline of Public Health Medicine, University of KwaZulu-Natal, Durban, South Africa; b Health Economics and HIV and AIDS Research Division (HEARD), University of KwaZulu-Natal, Durban, South Africa; c Centre for Rural Health, School of Nursing and Public Health, University of KwaZulu-Natal, Durban, South Africa; d Gender and Health Research Unit, South African Medical Research Council, Pretoria, South Africa; e Department of Psychology, University of Exeter, Exeter, UK; f KwaZulu-Natal Research Innovation and Sequencing Platform (KRISP), College of Health Sciences, University of KwaZulu-Natal, Durban, South Africa

**Keywords:** Emotional dysregulation, HIV, intimate partner violence, men, South Africa

## Abstract

Previous literature links emotional dysregulation (ED) to HIV acquisition risk and intimate partner violence (IPV) perpetration. This study assessed the relationship between ED, HIV acquisition risk, and IPV perpetration cross-sectionally and longitudinally among men (18–30 years) in urban informal settlements and rural areas in KwaZulu-Natal, South Africa. Data were drawn from 163 young men enrolled in a pilot randomized controlled trial of Stepping Stones and Creating Futures Plus (SSCF+). Regression models were used to examine baseline and longitudinal associations. Informed by previous findings that SSCF+ reduced ED among men with elevated depressive symptoms, we assessed whether the intervention modified ED – HIV/IPV associations in this subgroup using ED × intervention interaction terms among participants with elevated depressive symptoms (*n* = 56). Cross-sectionally, ED was associated with multiple sexual partners, alcohol use and drug use. Longitudinally, only alcohol abuse remained significantly associated with ED (adjusted odds ratio [aOR] 1.06, 95% CI 1.02–1.11). ED increased the risk of emotional and combined IPV perpetration cross-sectionally, and longitudinally ED was associated with physical (aOR 1.07, 95% CI 1.02–1.13), emotional (aOR 1.06, 95% CI 1.02–1.11), sexual (aOR 1.05, 95% CI 1.01–1.10), and combined IPV perpetration (adjusted beta coefficient [aβ] 0.16, 95% CI 0.03–0.34). Among men with elevated depressive symptoms, combined IPV perpetration increased with ED in the control group but remained relatively flat in the intervention group. Addressing ED within IPV and HIV prevention programming may be an important strategy for reducing men’s IPV perpetration and HIV risk.

## Introduction

South Africa has the largest HIV epidemic globally, with 7.6 million people estimated to be living with HIV (UNAIDS, 2022). Despite progress in HIV prevention and treatment, challenges persist, particularly among young people. In 2017, 88 000 new HIV cases were recorded in youth aged 15–25, accounting for almost 40% of total new acquisitions that year (South Africa National AIDS Council [AIDS], 2022; Zuma et al., 2022).

While HIV and social services programs support young women and girls due to their vulnerability to HIV (van der Wal et al., 2021), there are gaps in the response to HIV for men and boys (Mills et al., 2012). Factors increasing men’s HIV risk include sexual behaviours such as having multiple partners and unprotected intercourse (Cooperman et al., 2007; Huerga et al., 2017; Rugigana et al., 2015; Ssebunya et al., 2019). Alcohol and substance use also elevate HIV risk by impairing judgment and reducing condom use (Carels et al., 2022; Chersich & Rees, 2010; Cho & Yang, 2023; Doku, 2012; Lv et al., 2024; Mbuthia et al., 2020; Probst et al., 2018).

There is a strong correlation between HIV and intimate partner violence (IPV) (Rigby & Johnson, 2017). IPV includes physical, sexual, or emotional violence against an intimate partner (World Health Organization, 2021, 2022) and men are typically the perpetrators (World Health Organization, 2012). Men who commit sexual violence frequently do not use condoms (Davis et al., 2008) and forced penetration may cause injuries that increase HIV acquisition risk. Men who perpetrate violence are also more likely to engage in behaviours that elevate their own HIV risk, such as alcohol and drug use and having multiple sexual partners (Davis et al., 2012, 2018; Dunkle et al., 2006; Ramsoomar & Maker-Diedericks, 2021).

Men’s masculinities, particularly those shaped by inequitable gender attitudes, have been frequently linked to HIV acquisition risk behaviours and IPV perpetration (Fleming et al., 2016; McCarthy et al., 2018; Reidy et al., 2014; Sikweyiya et al., 2014; Sutton, 2024; Zeglin, 2015), and growing research suggests that men’s poor mental health also contributes to HIV acquisition risk and IPV perpetration. Evidence on the relationship between mental health and HIV acquisition risk among men remains limited, with most studies drawing on mixed-sex samples. A study in Limpopo, South Africa among university students, associated substance use, harmful alcohol use, and post-traumatic stress to HIV risk behaviours in men (Pengpid et al., 2013). Another study in the United States of America (U.S.A.) found that male sex, elevated depressive symptoms, and associated behaviours such as smoking and alcohol use were associated with elevated HIV risk (Xu et al., 2023). A Swedish study indicated that men with mental health disorders (e.g. depression) were more likely to perpetrate violence against women (Yu et al., 2019). While in Zimbabwe, depressive symptoms and post-traumatic stress were linked to men’s perpetration of IPV perpetration (Machisa & Shamu, 2018). Similarly, a pooled analysis of studies from South Africa, Ghana, Rwanda and the occupied Palestinian Territories found both depressive symptoms and post-traumatic stress symptoms increased the likelihood of men’s perpetration of IPV (Ramsoomar et al., 2021).

While various mental health conditions such as depression and post-traumatic stress have been linked to HIV acquisition risk and IPV perpetration, emotional dysregulation (ED) has not been extensively studied in relation to HIV and IPV in South Africa. ED is a transdiagnostic construct central to the maintenance of psychopathology (Sloan et al., 2017), and is associated with a range of mental health disorders, making it crucial in mental health research. ED refers to difficulties in managing emotions healthily, leading to responses outside the usual acceptable range, such as extreme anger and destructive actions (Tacchini & Vismara, 2019). ED encompasses several aspects, including trouble with goal-directed behaviour, a lack of clarity and awareness about one’s emotions, limited confidence in or access to effective emotion regulation strategies, challenges in controlling impulsive actions during distress and an inability to accept negative emotions (Gratz & Roemer, 2004; Wolff et al., 2019).

ED is associated with an increased risk of HIV acquisition behaviours, primarily in studies conducted in the U.S.A.. Among men and women in the U.S.A., ED was associated with sexual risk-taking with uncommitted partners, impulsive sexual behaviours, and intent to engage in risky sexual behaviours, as measured by the Sexual Risk Survey (Turchik & Garske, 2009; Weiss et al., 2019). Another study conducted in U.S.A. among justice-involved adolescents found that ED was associated with risky sexual behaviour (i.e. unprotected sex) (Miller et al., 2012). ED has also been suggested to contribute to compulsive sexual behaviours, representing a failure to cope with sexual impulses and resulting in uncontrolled sexual actions (Lew-Starowicz et al., 2020).

ED is also associated with an increased risk of IPV perpetration in studies from the U.S.A. and Canada. In the U.S.A., college men who struggled with ED were more likely to perpetrate violence against women (Gildner et al., 2021). Similarly, men with ED were found to more likely perpetrate dating violence (Shorey et al., 2015; Stappenbeck et al., 2016). A study with Canadian men also identified ED as mediating the relationship between attachment insecurities and IPV perpetration (Douadi et al., 2024).

The combined burden of poor mental health, HIV acquisition risk, and IPV perpetration among young men is often most pronounced in resource-constrained settings, such as rural regions and urban informal settlements in South Africa. These areas typically have limited economic opportunities and lack essential services like sewage systems and clean running water (du Toit, 2017; Wilna & Slabbert, 2010). Consequently, poverty levels are high, with food insecurity reported at 67.4% in a rural study (Tambe et al., 2023) and 65% in an urban informal settlement (Mkhize & Sibanda, 2022). Due to challenging living conditions, unemployment, and poverty, many men are likely to experience stress and poor mental health, especially given societal expectations for them to provide within relationships (Gibbs et al., 2014). The prevalence of poor mental health among men in urban informal settlements in South Africa was found to be high (46.8% depression and 14.4% post-traumatic stress) (Oyekunle, Tomita, et al., 2023). Studies also indicate high HIV prevalence in these communities (Gibbs, Reddy, et al., 2020) and HIV acquisition risk behaviours being common (Gibbs et al., 2019; Psaki et al., 2022). Men in these contexts are also more likely to perpetrate violence, with one study reporting that over half of the men (56.9%) perpetrated physical and/or sexual IPV in the past year (Gibbs et al., 2018; Gibbs, Dunkle, et al., 2020).

There is limited research on the relationship between ED, HIV acquisition risk behaviours, and IPV perpetration in Africa, including South Africa. Therefore, we sought to conduct a secondary analysis of data to assess the cross-sectional and longitudinal relationship between ED, HIV acquisition risk, and IPV perpetration among young men (18–30 years) in rural areas and urban informal settlements in South Africa.

## Methods

We conducted a secondary analysis of data from a pilot cluster randomized controlled trial of Stepping Stone and Creating Futures Plus (SSCF+) conducted in rural and urban informal settlements in eThekwini Municipality, KwaZulu-Natal (KZN). Of the 11.5 million reported to live in KZN, about 52.5% live in rural areas (Trade & Investment KwaZulu-Natal, 2020) while there are an estimated 587 informal settlements, accounting for almost 25% of the eThekwini Municipality population (Misselhorn, 2022).

This study focused on young people aged 18–30 who were not in full-time work or education, residing in the study areas, and able to communicate in English, isiZulu, or isiXhosa. Exclusions included individuals using alcohol or behaviour-altering drugs during recruitment or study activities, and those unable or unwilling to give informed consent.

Participants were recruited in single-gender friendship groups to form clusters. Eligible individuals were identified and asked to recruit up to 10 friends who also met the criteria. A total of 163 young men were enrolled and clusters were randomly assigned to either the intervention or delayed control group using an Excel random number generator, with equal distribution (1:1). The SSCF+ intervention consisted of 15 sessions, each approximately 3 hours long, addressing gender inequalities, challenging violence in relationships, strengthening livelihoods, and promoting well-being. A detailed description of SSCF+ is reported elsewhere (Gibbs et al., 2025).

Data were collected at baseline (May – June 2023, before randomization) and at follow-up approximately 5 months later (October – November 2023). The control group received the intervention after end-line data collection. Data were collected using self-administered questionnaires on cell phones through the KoboToolbox platform, with audio support available in English, isiZulu, or isiXhosa, and a fieldworker nearby for assistance.

### Measures

#### Outcome variables

HIV acquisition risk behaviours and intimate partner violence perpetration were the main outcomes for this study.

#### HIV acquisition risk behaviour

##### Condom Use

Participants were asked whether they used condoms in their last sexual encounter, with responses of yes or no.

##### Condom Use Frequency

Participants were asked how often they used condoms in the last 6 months, with responses coded as 1 = never, 2 = sometimes, 3 = often, and 4 = always. The responses were recoded into two categories: 1=‘never’ and 2, 3, and 4 as ‘sometimes or always’.

##### Number of Sexual Partners

Participants reported the number of main partners, khwapeni (other partners besides the main partner), and people they had sex with in the past six months. Responses were combined into one score: ‘number of sexual partners in the past six months’.

##### Drug Use

Participants were asked about drug use or substances that made them high in the past six months, with responses of 0 = never, 1 = once, and 2 = more than once. The responses were recoded into two categories: 0 = never and 1/2 = at least once.

##### Alcohol Use

Participants completed three questions adapted from the Alcohol Use Disorders Identification Test (AUDIT) by the (World Health Organization, 2001). The questions assessed the frequency of alcohol consumption in the past 6 months, with varying responses per question, all with 5 options. A composite score ranging from 0–10 was obtained and recoded into two categories: 0/3 = no alcohol problem and 4/10 = alcohol problem.

#### Intimate partner violence perpetration

IPV perpetration was assessed using scales from the World Health Organization (WHO) Violence Against Women adapted for men’s perpetration in South Africa (Jewkes et al., 2014) which captures behaviourally specific acts of physical, emotional, sexual, and economic violence. Participants were asked a series of items about whether they had perpetrated IPV (i.e. physical, emotional, sexual, and economic) in the past 6 months, with the ‘target’ being their current or ex-partner. Specific scales are below.

##### Physical IPV perpetration

Participants answered five behaviourally specific questions about perpetrating physical violence, such as slapping, hitting, pushing, or using a knife on their intimate partner in the past 6 months.

##### Emotional IPV perpetration

Participants answered five questions about psychological abuse and threats, such as insulting, threatening, belittling, and damaging items of importance to their intimate partners in the past 6 months.

##### Sexual IPV perpetration

Participants answered three questions related to sexual coercion, such as forcing or threatening an intimate partner to have sexual intercourse in the past 6 months.

##### Economic IPV perpetration

Five questions were used to assess economic IPV, such as stopping a partner from going to work, taking a partner’s earnings against her will, and throwing the partner out of the house in the past 6 months.

All items about IPV perpetration (i.e. physical, emotional, sexual, and economic) had a 4-point response: ‘never’, ‘once’, ‘a few times’, and ‘many times’. Outcomes were dichotomized for each form of IPV, with those who never perpetrated IPV recoded as no and those who had perpetrated recoded as yes.

##### Combined IPV perpetration

All forms of IPV (physical, emotional, sexual, and economic) were combined to create a composite IPV score. Responses across the four IPV types were summed to generate a continuous measure, with higher scores indicating greater overall IPV perpetration. Unlike the individual IPV outcomes, the combined IPV variable was not dichotomised.

### Exposure variables

The primary exposure variable of the study was emotional dysregulation, assessed using the Difficulties in Emotional Regulation Scale-16 (DERS-16) (Bjureberg et al., 2016). This scale has been confirmed as both valid and reliable across various contexts (Demirpence Secinti & Sen, 2023; Fekih-Romdhane et al., 2023), including South Africa (Ward-Smith et al., 2024). The DERS-16 consists of 16 items evaluating how individuals manage their emotions when distressed, with responses recorded on a 5-point Likert scale: 1 = almost never, 2 = sometimes, 3 = about half the time, 4 = most of the time, and 5 = almost always. Scores were summed to produce a composite variable ranging from 1 to 80. The Cronbach alpha was 0.91.

Data on sociodemographic variables, including age, education level, and relationship status, were also gathered. Participants’ ages ranged from 18 to 30 years and were recoded into three categories: 18 to 20 years, 20 to 24 years, and 25 to 30 years. Highest education levels reached ranged from grade 1 to 12 and were recoded into two categories: grades 1–11 as primary to high school and grade 12 as final high school year. Relationship status was assessed as either in a relationship or not.

Gender attitudes were included as a covariate, given well-established evidence linking gender-inequitable attitudes to both HIV acquisition risk and IPV perpetration in men. These attitudes were measured using items adapted from the Gender-Equitable Men (GEM) scale (Pulerwitz & Barker, 2008), which assesses individual-level gender-related and gender-inequitable beliefs rather than broader social norms with responses on a four-point Likert scale (1 = strongly disagree to 4 = strongly agree). Responses were combined into a composite score, where higher scores indicated less gender-equitable attitudes.

### Data analysis

We summarized baseline sociodemographic and clinical characteristics by intervention arm using descriptive statistics. For categorical variables, we provided frequencies and percentages. For continuous variables, we reported means and standard deviations. To assess the success of randomization, we compared the sociodemographic and clinical characteristics of the two groups using chi-square tests for categorical variables and linear regression for continuous variables.

To examine the cross-sectional relationship between ED, HIV acquisition risk, and IPV perpetration, we fitted binary logistic regression models for dichotomous outcomes and linear regression models for continuous outcomes using baseline data. All models were adjusted for gender norms. Additionally, we further adjusted the models(referred to as adjusted models) for age, education status, and relationship status. Unadjusted models are reported in the tables for reference.

We conducted various longitudinal analyses to examine the relationship between ED, HIV acquisition risk, and IPV perpetration.

First, we assessed changes in HIV acquisition risk, IPV perpetration, and ED over time within the intervention and control groups, using mean differences for continuous variables and percentage point differences for binary variables.

Second, to evaluate the longitudinal relationship between ED and HIV acquisition risk/IPV perpetration, we regressed baseline ED onto end-line HIV acquisition risk factors and IPV perpetration outcomes We adjusted all longitudinal models for baseline outcomes, intervention status, gender attitudes and increase in ED (ED at end-line minus ED at baseline). Additionally, we further adjusted the models for age, education status, and relationship status – referred to as our adjusted models while results from unadjusted models are reported in the tables for reference. We also report change in ED in addition to gender norms as a main covariate in the tables as it may provide insights into how shifts in emotional regulation influence its relationship with HIV acquisition risk and IPV perpetration over time.

Finally, given previous findings that the SSCF+ intervention reduced ED among men with elevated depressive symptoms (Nyoni et al., 2025b), subgroup analyses were conducted only among men in this category. This subgroup analysis examined whether the intervention modified the relationship between ED and HIV acquisition risk/IPV perpetration. To do so, we incorporated an interaction term (ED × intervention) into our longitudinal models among men with elevated depressive symptoms (*n* = 56). All regression models in this interaction analysis were adjusted for baseline outcome variables, gender attitudes and increase in ED, while our adjusted models also included controls for age, education status, and relationship status. Joint significance (of the main effects and their interaction terms) as well as interaction term alone was assessed using Wald tests applied to the adjusted regression models. Since interaction effects can be complex to interpret, we used post-estimation graphs to visualize the findings and reported linear combination (lincom) estimates to quantify differences in IPV levels between intervention arms at various ED levels. Because the parent trial was a pilot study, the subgroup analysis was not powered to detect small or moderate effects; therefore, these results should be interpreted as exploratory and hypothesis-generating. All regression models accounted for the cluster design and survey weights using survey adjusted estimation and analyses were conducted in STATA 16.

### Ethical consideration

The SSCF+ was approved by South Africa Medical Research Council (EC023-10/2022) and the University of Exeter (570602) ethics committees. The trial was pre-registered at clinicaltrials.gov (NCT05783336). Our study (i.e. the use of SSCF+ data) was approved by University of KwaZulu-Natal Biomedical Research Ethics Committee (BREC/00004912/2022). Participants consented fully in form of writing prior to participation in the study. All participants provided written informed consent prior to participation. Data were collected using self-completed questionnaires on mobile devices, which reduced the need for verbal disclosure of sensitive behaviours, including IPV perpetration. Fieldworkers were trained to monitor participant distress, and a social worker was available for referral should participation cause discomfort or distress. These procedures were designed to minimise potential harm and ensure participant safety.

## Results

A total of 163 men were recruited and 84 (51.53%) were randomly assigned in the intervention groups and 79 (48.47%) men assigned to the control group. Only five participants were lost to follow up, leading to a retention rate of 96.93% at end-line.

### Baseline sociodemographic and clinical characteristics

#### Overall baseline sociodemographic and clinical characteristics

Half of the participants (52.76%) were aged 20–24 years, and almost half (49.08%) reported that they completed their final year of high school. Most participants reported being in an intimate relationship (86.88%). Almost half of the participants did not use condoms during their last sex (48.41%) and the mean number of their sexual partners in the past 6 months being 6.67 (SD = 6.04). Just above 40% had used drugs in the past 6 months and almost 30% had an alcohol problem. Past 6 month IPV perpetration was high, the most perpetrated violence being emotional violence (57.06%). The mean score for ED was 30.01 (SD = 10.81), the minimum and maximum response scores being 15 and 68 respectively (Table 1).

#### Baseline sociodemographic and clinical characteristics by intervention arm

At baseline there were no significant differences between the intervention arms except for past 6 month economic IPV perpetration (Table 1).

#### The relationship between ED, and HIV acquisition risk and IPV perpetration at baseline

##### The relationship between ED, and HIV acquisition risk at baseline

Cross-sectionally, in the adjusted models (Table 2), ED was positively associated with having more sexual partners (adjusted beta coefficient [aβ] = 0.11, 95% CI: 0.02–0.21), increased drug use (adjusted odds ratio [aOR] = 1.07, 95% CI: 1.03–1.11), and having an alcohol problem (aOR = 1.09, 95% CI: 1.04–1.13). Gender norms were not associated with any of the HIV acquisition risk factors.

##### The relationship between ED and IPV perpetration at baseline

Cross-sectionally, in the adjusted models (Table 2), ED was associated with emotional IPV perpetration (aOR = 1.05, 95% CI: 1.00–1.10) and combined IPV perpetration (aβ = 0.17, 95% CI: 0.06–0.29). Gender norms were associated with physical IPV perpetration (aOR = 1.11, 95% CI: 1.03–1.19).

### Longitudinal comparison of outcomes and ED within the arms over time

#### Longitudinal comparison of HIV acquisition risk within SSCF+ and control arms

Between baseline and end-line (Table 3) there was a significant increase in the percentage of men who used drugs in the SSCF+ groups by 13.98% points. All the other differences were not statistically significant.

#### Longitudinal comparison of IPV perpetration within SSCF+ and control arms

There was a significant increase of physical IPV perpetration and combined IPV perpetration by 14.26 and 2.14% points respectively in the control groups. All the other differences were not statistically significant.

#### Longitudinal comparison of ED within SSCF+ and control arms

There was no significant differences between ED in all groups.

### The longitudinal relationship between ED, HIV acquisition risk and IPV perpetration

#### Multivariable longitudinal relationship between ED and HIV acquisition risk

The longitudinal analysis (Table 4) found that, baseline ED was associated with having an alcohol problem at end-line in the adjusted model (aOR = 1.06, 95% CI: 1.02–1.11).

An increase in ED over time was associated with having more sexual partners (aβ = 0.07, 95% CI: 0.03–0.14). Gender attitudes were not associated with any of the HIV acquisition risk factors.

#### Multivariable longitudinal relationship between ED, and IPV perpetration

Baseline ED was associated with physical IPV perpetration (aOR = 1.07, 95% CI: 1.02–1.13), emotional IPV perpetration (aOR = 1.06, 95% CI: 1.02–1.11), sexual IPV perpetration (aOR = 1.05, 95% CI: 1.01–1.10), and combined IPV perpetration (aβ = 0.16, 95% CI: 0.03–0.34) at end-line. An increase in ED over time was associated with physical IPV perpetration (aOR = 1.10, 95% CI: 1.03–1.18), sexual IPV perpetration (aOR = 1.07, 95% CI: 1.03–1.12), and combined IPV perpetration (aβ = 0.19, 95% CI: 0.05–0.37). Gender attitudes were associated with emotional IPV perpetration (aOR = 1.11, 95% CI: 1.03–1.21) and combined IPV perpetration (aβ = 0.29, 95% CI: 0.05–0.37).

### The interaction between ED and intervention status on HIV acquisition risk and IPV perpetration among men with elevated depressive symptoms

#### The interaction between ED and intervention status on HIV acquisition risk among men with elevated depressive symptoms

Wald tests for joint significance indicated that ED, intervention status, and their interaction were collectively associated with substance use outcomes, with statistically significant joint effects observed for drug use (χ^2^ (3)=9.90, *p* = 0.019) and alcohol problems (χ^2^ (3)=11.95, *p* = 0.008). Interaction terms were not statistically significant for alcohol problems. For drug use, the ED × intervention interaction term was statistically significant in adjusted models only, and this was further supported by a Wald test of the interaction term alone (χ^2^ (1)=4.09, *p* = 0.04; Table 5).

#### The interaction between ED and intervention status on IPV perpetration among men with elevated depressive symptoms

**Figure 1:**
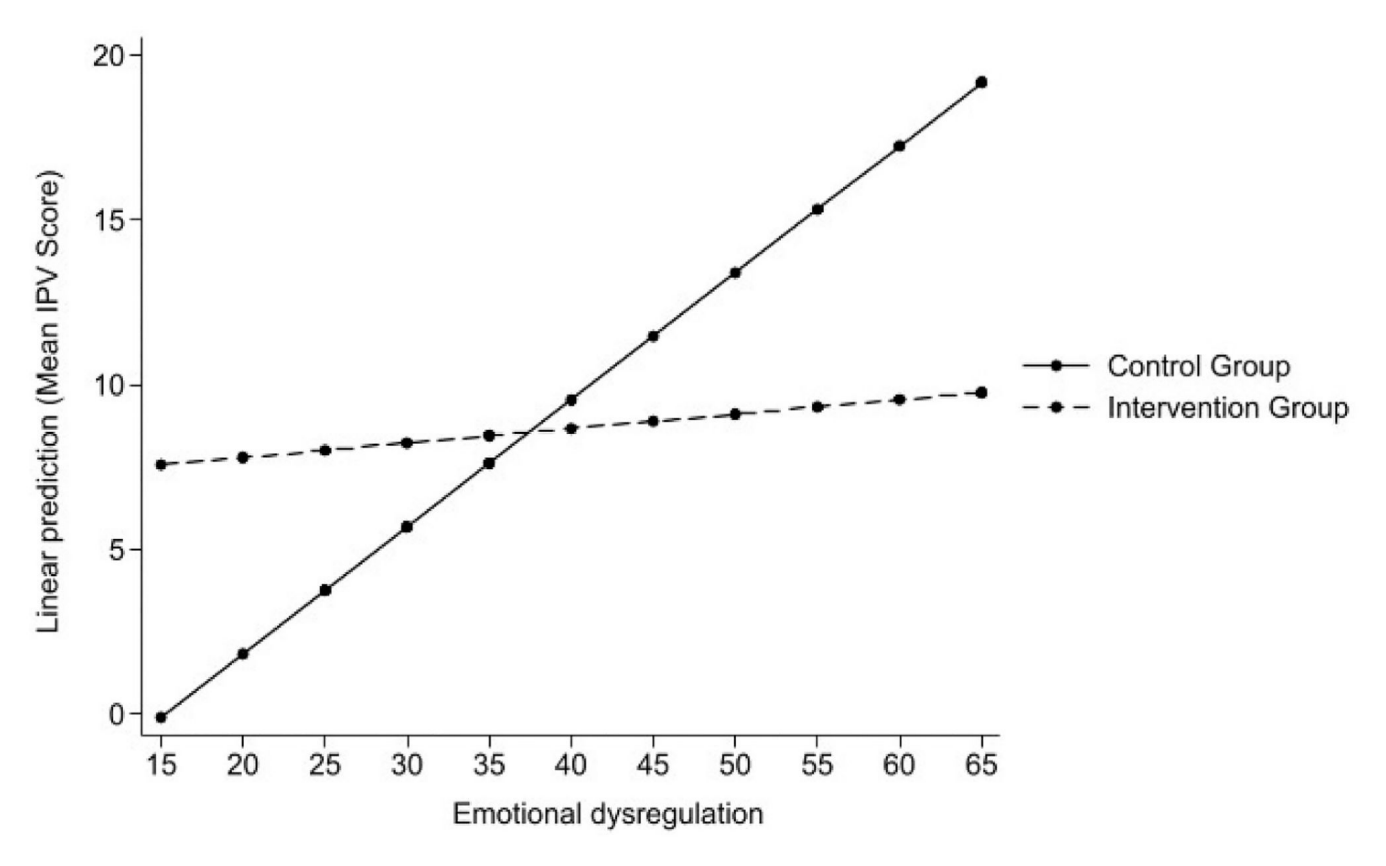
Graphical representation of the interaction between ED and the intervention against combined IPV perpetration.

Wald tests for joint significance of the ED, intervention status, and their interaction were statistically significant for combined IPV perpetration outcome (F (3,26)=3.04, *p* = 0.047). Evidence of effect modification by intervention status for combined IPV perpetration was further supported by a Wald test of the interaction term alone (F(1,26)=5.23, *p* = 0.03), consistent with the statistically significant interaction observed in the regression models (Table 5). No statistically significant association was detected in the other IPV outcomes (Table 5). Post-estimation plots (Figure 1) showed that combined IPV perpetration increased with higher levels of ED in the control group, while the association remained relatively flat in the intervention group. Linear combination analyses indicated that combined IPV perpetration scores were significantly higher in the control group compared with the intervention group at high levels of ED scores of 60 (β = 7.26, 95% CI: 0.21–14.31) and 65 (β = 8.91, 95% CI: 0.59–17.23).

### Discussion

This study examined the relationship between ED and HIV acquisition risk and IPV perpetration among young men in rural areas and urban informal settlements in KwaZulu-Natal. We identified two major findings. First, ED increased HIV acquisition risk and IPV perpetration even after adjusting for a key covariate, gender attitudes. Second, among men with elevated depressive symptoms, we found that the effect of the intervention on the relationship between ED and IPV perpetration differed by study arm. Specifically, at high levels of ED, IPV perpetration was higher among men in the control group, whereas lower IPV perpetration was observed among those in the intervention group. This pattern suggests that the intervention may hold promise in mitigating the effects of ED among men with elevated depressive symptoms.

We found that ED was significantly associated with HIV acquisition risk factors both cross-sectionally and prospectively. However, the number of risk factors associated with ED decreased at end-line, with alcohol use remaining the only persistent correlate. This may reflect alcohol’s role as a social accepted and accessible coping mechanism in these contexts. Additionally, an increase in ED over time was associated with reporting more sexual partners. Previous work has similarly linked ED to HIV acquisition risk behaviours, with several ED dimensions such as impulsivity, promoting behaviours that increase HIV risk (Curry et al., 2018). Ineffective emotion regulation strategies, such as avoidance coping (Aldao et al., 2014) may also lead individuals to numb distress through alcohol and substance use (Danielson et al., 2024; van der Heijden et al., 2022), and both have been shown to increase engagement in risky sex (Lv et al., 2024; Probst et al., 2018) which is a risk factor of HIV acquisition. Other studies report that some men cope with negative emotions through sex, sometimes in risky contexts (Ellis & Orcutt, 2023; Martin & Alessi, 2010).

IPV perpetration was associated with ED both cross-sectionally and prospectively, suggesting a strong and enduring relationship. All forms of IPV perpetration, except emotional IPV perpetration, were also associated with increase in ED, indicating that worsening emotional dysregulation may heighten the likelihood of physical, sexual, and economic violence perpetration. This aligns with research showing that ED can escalate interpersonal conflict and aggression (Navas-Casado et al., 2023; Roberton et al., 2012). Emotional dysregulation was also identified to increase the risk of IPV perpetration in other studies in U.S.A. and Canada (Lebeau et al., 2025; Shorey et al., 2015; Stappenbeck et al., 2016). Mechanisms through which ED might result in violence perpetration include via impulsive behaviours which is one of the dimensions of ED. Individuals who tend to be impulsive tend to perpetrate violence either verbally or physically (Bresin, 2019; Gildner et al., 2021). Emotional IPV perpetration, however, was not associated with increases in ED. This may be because emotional IPV is one of the highly prevalent form of violence as evidenced by a South African National Gender-Based violence study (Human Sciences Research Council, 2025). Its high prevalence suggests that emotional IPV may reflect a common, routine behavioural pattern within relationships, making it more persistent and less likely to shift in response to short-term changes in ED. Consistent with this, emotional IPV perpetration was also the most prevalent form of IPV perpetration in our sample.

A key finding in our study was that among men with elevated depressive symptoms, IPV perpetration is significantly lower in the intervention group compared to the control group when ED is particularly pronounced. The mechanism behind this protective effect of the intervention likely stems from the intervention’s ability to reduce ED in men with elevated depressive symptoms, which in turn may have led to a decrease in IPV perpetration. Prior research (Oyekunle, Gibbs, et al., 2023) found that improvements in depressive symptoms mediated reduced IPV perpetration following an intervention, suggesting that addressing mental health may be a critical pathway through which interventions disrupt the poor mental health – IPV cycle. Evidence of effect modification for drug use emerged only after covariate adjustment, suggesting that adjustment for age, education and relationship status may have reduced residual confounding and clarified the conditional association between ED and drug use across intervention arms. In contrast to combined IPV perpetration, where interaction effects were robust across model specifications, the adjusted-only interaction observed for drug use should be interpreted cautiously, particularly given the pilot nature of the trial and limited power to detect interaction effects.

### Limitations

This study has several limitations. Firstly, the sample size was relatively small and the follow-up period was short, which limits the generalisability of the findings and restricts our ability to examine longer-term patterns of ED and HIV-acquisition risk and IPV perpetration.

Secondly, although the WHO violence against women scale captures behaviourally specific acts of IPV perpetration, ED was measured primarily as an affective – cognitive construct. The study did not include a separate measure of general behavioural regulation outside IPV-specific actions which limits our ability to describe the behavioural pathways through which ED may escalate into IPV perpetration.

Thirdly, the current study did not include measures capturing the relational contexts through which IPV occurs, such as relationship quality, communication patterns, relationship duration, and coercive control. While these factors are important for understanding how and why IPV unfolds within relationships, the present study was designed to quantitatively examine the association between ED and IPV perpetration rather than broader relationship processes. However, our prior in-depth qualitative study (Nyoni et al., 2025a) explored these relational dynamics in detail, providing complementary insights that extend the interpretation of the current findings.

### Implications

Despite these limitations, our findings have important implications, particularly in settings with high IPV and HIV prevalence. First, although based on a small sample and a short follow-up period, this study provides preliminary evidence that can inform future research and intervention development. Larger studies with adequate power for subgroup analyses are recommended to build on these preliminary findings. Future research should also explore these relationships over longer periods to better understand how changes in ED influence HIV acquisition risk and IPV perpetration over time. In addition, future studies would benefit from incorporating expanded IPV measures, including coercive control and other dimensions of relational power, as well as indicators of relationship quality and dynamics, to provide a more comprehensive understanding of how ED operates within intimate relationships.

Second, our results highlight ED as a critical risk factor for both HIV acquisition risk and IPV perpetration among young men in resource-limited environments. Interventions seeking to prevent men’s perpetration of IPV should consider addressing ED, as incorporating emotion regulation components may strengthen their effectiveness in reducing IPV. Strengthening mental health support within HIV and IPV programming may also offer a promising pathway to reducing both IPV perpetration and HIV risk.

## Conclusion

Emotional dysregulation is a significant risk factor for both HIV acquisition risk and IPV perpetration among young men in resource-constrained settings in South Africa. Addressing ED is therefore essential for reducing IPV perpetration and the associated HIV risks. As ED is a transdiagnostic mental health construct that underpins a range of psychological difficulties, integrating mental health components, particularly those focused on improving emotion regulation into IPV and HIV prevention programs may strengthen their overall effectiveness and better reduce harm among young men.

## Figures and Tables

**Table 1:** Overall baseline sociodemographic and clinical characteristics by intervention arm.

	Overall	SSCF+	Control	P-value
	**N = 163**	**N = 84**	**N = 79**	
Socio-demographics	**n(%)**	**n(%)**	**n(%)**	
Age				
<20 years	12(7.36)	5(5.95)	7(8.86)	
20–24 years	86(52.76)	39(46.43)	47(59.49)	
≥25 years	65(39.88)	40(47.62)	25(31.65)	0.088
Education level				
Primary to high school	83(50.92)	39(46.43)	44(55.70)	
Final high school year	80(49.08)	45(53.57)	35(44.30)	0.217
Relationship				
In a relationship	139(86.88)	67(82.72)	72(91.14)	
Single	21(13.12)	14(17.28)	7(8.86)	0.118
HIV risk related factors				
Condom use during last sex				
No	76(48.41)	44(55.00)	32(41.56)	
Yes	81(51.59)	36(45.00)	45(58.44)	0.080
Condom use frequency ≤6 months				
Never	27(17.31)	12(15.00)	15(19.74)	
Sometimes/always	129(82.69)	68(85.00)	61(80.26)	0.430
Drug use				
Never	95(58.64)	47(55.95)	48(61.54)	
At least once				
Alcohol problem	67(41.36)	37(44.05)	30(38.46)	0.470
No	114(70.37)	59(71.08)	55(69.62)	0.844
Yes	48(29.63)	24(28.92)	24(30.38)	0.605
Number of sexual partners	**Mean (SD)**	**Mean (SD)**	**Mean (SD)**	
	6.67(6.04)	6.92(6.05)	6.42(6.00)	
IPV perpetration	**n(%)**	**n(%)**	**n(%)**	
Physical IPV				
No	109(66.87)	52(61.90)	57(72.15)	
Yes	54(33.13)	32(38.10)	22(27.85)	0.169
Emotional IPV				
No	70(42.94)	31(36.90)	39(49.37)	
Yes	93(57.06)	53(63.10)	40(50.36)	0.115
Sexual IPV				
No	131(80.86)	66(78.57)	65(83.33)	
Yes	31(19.14)	18(21.43)	13(16.67)	0.421
Economic IPV				
No	84(51.53)	36(42.86)	48(60.76)	
Yes	79(48.47)	48(57.14)	31(39.24)	0.031*
	**Mean (SD)**	**Mean (SD)**	**Mean (SD)**	
Combined IPV	4.66(6.16)	5.21(6.44)	4.06(5.79)	0.239
Emotional dysregulation	30.01(10.81)	30.12(10.36)	29.90(11.27)	0.895

* statistically significant, () in % unless otherwise specified, SD = standard deviation, Condom use frequency ≤6 months

frequency of using condoms in the past or equal to 6 months, combined IPV = all the types of IPV combined (i.e. physical, emotional, sexual and economic).

**Table 2:** Emotional dysregulation and its association with HIV acquisition risk and IPV perpetration at baseline (*N* = 163).

Variables	Unadjusted OR(95% CI)	Unadjusted p-value	Adjusted OR(95% CI)	Adjusted p-value
HIV risk related				
Condom use during last sex	.98(.95–1.01)	0.119	.97(.94–1.00)	0.051
ED	1.00(.95–1.07)	0.880	.99(.93–1.06)	0.850
Gender attitudes				
Condom use frequency ≤6	.98(.94–1.02)	0.377	.98(.94–1.03)	0.424
months	.98(.90–1.06)	0.546	.99(.91–1.07)	0.809
ED	1.06(1.02–1.10)	0.001*	1.07(1.03–1.11)	<0.001*
Gender attitudes	1.00(.94–1.07)	0.883	.99(.92–1.05)	0.676
Drug use	1.07(1.04–1.11)	<0.001*	1.09(1.04–1.13)	<0.001*
ED	.98(.91–1.03)	0.269	.95(.90–1.01)	0.109
Gender attitudes				
Alcohol problem				
ED				
Gender attitudes				
Number of sexual partners	**Unadjusted β(95% CI)**	**Unadjusted *p*–value**	**Adjusted β(95% CI)**	**Adjusted *p*–value**
ED				
Gender attitudes	.10(.01–.19)	0.035*	.11(.02–.21)	0.019*
IPV perpetration	.02(–.22–.26)	0.892	.04(–.16-24)	0.695
	**Unadjusted OR (95% CI)**	**Unadjusted *p*-value**	**Adjusted OR (95% CI)**	**Adjusted *p*-value**
Physical IPV	1.02(.99–1.06)	0.126	1.03(.99–1.06)	0.105
ED	1.09(1.01–1.17)	0.020*	1.11(1.03–1.19)	0.008*
Gender attitudes				
Emotional IPV	1.05(1.01–1.09)	0.017*	1.05(1.00–1.10)	0.022*
ED	1.01(.95–1.07)	0.753	1.02(.96–1.09)	0.528
Gender attitudes				
Sexual IPV	1.03(.99–1.03)	0.104	1.02(.98–1.07)	0.234
ED	1.07(.98–1.18)	0.114	1.06(.95–1.17)	0.284
Gender attitudes				
Economic IPV	1.01(.98–1.05)	0.376	1.01(.98–1.05)	0.323
ED	1.06(.99–1.13)	0.099	1.06(.99–1.13)	0.111
Gender attitudes				
	**Unadjusted β(95% CI)**	**Unadjusted *p*-value**	**Adjusted β(95% CI)**	**Adjusted *p*-value**
Combined IPV	.17(.05–.28)	0.005*	.17(.06–.29)	0.004*
ED	.10(–.07–.27)	0.242	.09(–.09–.28)	0.326
Gender attitudes				

* statistically significant *p* < 0.05, ED = emotional dysregulation, OR = odds ratio, β = coefficient, CI = confidence interval. Gender attitudes was a covariate for both models and the adjusted models were further adjusted for age, education status and relationship status.

**Table 3:** Change in HIV acquisition risk factors, IPV perpetration factors and emotional dysregulation from baseline to end-line (*N* = 163).

Variables	Baseline		End-line		Baseline End-line			
HIV risk related factors	SSCF+(*n* = 84) n(%)	SSCF+(*n* = 82) n(%)	Percentage point difference over time	P-value	Control(*n* = 79) n(%)	Control(*n* = 76) n(%)	Percentage point difference over time	P-value
Condom use during last sex	44(55.00)	39(52.00)	–3.00	0.627	32(41.56)	30(45.45)	3.89	0.607
No								
Condom use frequency <6 months	12(15.00)	18(24.00)	9.00	0.086	15(19.74)	11(17.19)	–2.55	0.672
Never								
Drug use	37(44.05)	47(58.02)	13.98	0.024*	30(38.46)	34(44.74)	6.28	0.331
At least once								
Alcohol problem	24(28.92)	26(32.10)	3.18	0.658	24(30.38)	28(36.84)	6.46	0.261
Yes								
	**Mean(SD)**	**Mean(SD)**	**Difference in means over time**	**P-value**	**Mean(SD)**	**Mean(SD)**	**Difference in means over time**	**P-value**
Number of sexual partners	6.97(6.09)	6.12(4.90)	–0.85	0.232	6.47(6.09)	5.72(4.42)	–0.75	0.298
IPV perpetration past 6 months	**Baseline SSCF+**	**End-line SSCF+**	**Percentage point difference over time**	**P-value**	**Baseline Control**	**End-line Control**	**Percentage point difference over time**	**P-value**
Physical IPV	32(38.10)	25(30.86)	–7.24	0.175	22(27.85)	32(42.11)	14.26	0.013*
Yes								
Emotional IPV	53(63.10)	43(56.58)	–6.52	0.335	40(50.63)	39(57.35)	6.72	0.389
Yes								
Sexual IPV	18(21.43)	17(20.73)	–0.70	0.896	13(16.67)	17(22.37)	5.70	0.262
Yes								
Economic IPV	48(57.14)	37(48.68)	–8.46	0.176	31(39.24)	36(52.94)	13.70	0.054
Yes								
	**Mean(SD)**	**Mean(SD)**	**Difference in means over time**	**P-value**	**Mean(SD)**	**Mean(SD)**	**Differences in means over time**	**P-value**
Combined IPV	5.29(6.51)	6.56(8.94)	1.27	0.130	4.08 ((5.86))	6.22(8.56)	2.14	0.016*
Emotional dysregulation	30.18(10.50)	29.85 (11.44)	–0.33	0.814	29.95 (11.51)	30.47(9.94)	0.52	0.686

* statistically significant *p* < 0.05, SD = standard deviation.

**Table 4:** Emotional dysregulation and its longitudinal association with HIV acquisition risk and IPV

Variables	OR(95%CI)	P-value	aOR (95%CI)	P-value
**HIV risk related**				
Condom use during last sex				
ED	.97(.93–1.01)	0.138	.96(.93–1.01)	0.065
Change in ED	1.00(.97–1.04)	1.000	1.00(.97–1.04)	0.938
Gender attitudes	1.07(.99–1.16)	0.097	1.06(.98–1.16)	0.156
Condom use frequency <6 months				
ED	1.00(.96–1.05)	0.838	1.00(.96–1.05)	0.802
Change in ED	1.01(.96–1.06)	0.900	1.01(.96–1.07)	0.727
Gender attitudes	1.10(.97–1.25)	0.134	1.09(.95–1.24)	0.210
Drug use				
ED	1.04(1.00–1.09)	0.080	1.04(1.00–1.10)	0.089
Change in ED	1.04(1.00–1.09)	0.033*	1.04(1.00–1.08)	0.064
Gender attitudes	1.02(.96–1.08)	0.598	1.04(.97–1.11)	0.304
Alcohol problem				
ED	1.05(1.00–1.09)	0.030*	1.06(1.02–1.11)	0.005*
Change in ED	1.03(1.00–1.06)	0.062	1.03(1.00–1.06)	0.074
Gender attitudes	1.00(.92–1.08)	0.991	1.02(.93–1.11)	0.743
	**β(95%CI)**	**P-value**	**aβ(95%CI)**	**P-value**
Number of sexual partners				
ED	.07(–.01-.16)	0.088	.06(–.01-.16)	0.149
Change in ED	.07(.01–.13)	0.023*	.07(.02–.14)	0.018*
Gender attitudes	.13(.02–.25)	0.028*	.11(–.01-.22)	0.072
**IPV perpetration**	**OR(95%CI)**	**P-value**	**aOR (95%CI)**	**P-value**
Physical IPV				
ED	1.07(1.02–1.13)	0.006*	1.07(1.02–1.13)	0.010*
Change in ED	1.10(1.03–1.17)	0.003*	1.10(1.03–1.18)	0.007*
Gender attitudes	1.03(.96–1.12)	0.385	1.06(.96–1.15)	0.240
Emotional IPV				
ED	1.06(1.03–1.11)	0.005*	1.06(1.02–1.11)	0.024*
Change in ED	1.07(1.01–1.13)	0.042*	1.06(1.00–1.13)	0.100
Gender attitudes	1.10(1.03–1.18)	0.005*	1.11(1.03–1.21)	0.006*
Sexual IPV				
ED	1.05(1.01–1.10)	0.026*	1.05(1.01–1.10)	0.028*
Change in ED	1.08(1.03–1.13)	0.001*	1.07(1.03–1.12)	0.002*
Gender attitudes	1.02(.93–1.12)	0.708	1.04(.93–1.16)	0.473
Economic IPV				
ED	1.03(1.00–1.07)	0.131	1.03(1.00–1.08)	0.169
Change in ED	1.05(1.01–1.10)	0.024*	1.05(1.00–1.10)	0.056
Gender attitudes	1.07(.99–1.15)	0.092	1.07(.98–1.17)	0.119
	**β(95%CI)**	**P-value**	**aβ(95%CI)**	**P-value**
Combined IPV				
ED	.16(.02–.33)	0.043*	.16(.03–.34)	0.040*
Change in ED	.23(.06–.42)	0.010*	.19(.05–.37)	0.017*
Gender attitudes	.19(–.04–.43)	0.099	.29(.09–.50)	0.007*

* statistically significant *p* < 0.05, ED = emotional dysregulation, OR = odds ratio, β = coefficient, CI = confidence interval. Increase in ED over time, gender attitudes, baseline outcomes and the baseline intervention were covariates in both models while adjusted models were further adjusted for age, education status and relationship status.

**Table 5:** The impact of interacting ED with the intervention on HIV acquisition risk and IPV perpetration outcomes at end-line among men at risk of depression (*N* = 56).

Variables	OR(95%CI)	P-value	aOR(95%CI)	P-value
**HIV risk related**				
Condom use during last sex				
ED	.94(.86–1.03)	0.187	.95(.86–1.05)	0.337
Intervention	1.61(.01–281.84)	0.877	2.56(.01–508.00)	0.728
ED##Intervention	1.01(.89–1.14)	0.852	1.00(.87–1.14)	0.957
Gender attitudes	1.20(.98–1.47)	0.074	1.21(.96–1.51)	0.102
Condom use frequency ≤6 months				
ED	.97(.82–1.14)	0.699	.97(.80–1.17)	0.766
Intervention	.16(.00–127.89)	0.593	.31(.00–782.52)	0.772
ED##Intervention	1.05(.90–1.22)	0.559	1.02(.86–1.23)	0.771
Gender attitudes	1.21(1.04–1.41)	0.015*	1.27(1.00–1.62)	0.046*
Drug uset¶,§	1.12(1.03–1.21)	0.012*	1.12(1.03–1.23)	0.010*
ED	13.62(.29–643.22)	0.184	17.15(.43–679.92)	0.130
Intervention	.92(.84–1.01)	0.086	.91(.83–1.00)	0.043*
ED##Intervention	1.00(.90–1.12)	0.986	1.04(.95–1.14)	0.344
Gender attitudes	1.06(.93–1.20)	0.389	1.18(1.00–1.38)	0.046*
Alcohol problem¶	5.62(.01–4103.06)	0.608	37.32(.01–166150.20)	0.398
ED	.95(.81–1.11)	0.511	.89(.75–1.06)	0.204
Intervention	1.04(.93–1.16)	0.499	.93(.82–1.05)	0.236
ED##Intervention				
Gender attitudes				
	**β(95%CI)**	**P-value**	**aβ(95%CI)**	**P-value**
Number of sexual partners				
ED	.00(–.13-.14)	0.324	.03(–.11-.16)	0.680
Intervention	–2.33(–9.23–4.57)	0.494	–1.01(–8.41–6.40)	0.782
ED##Intervention	.11(–.08-.30)	0.259	.07(–.12-.27)	0.461
Gender attitudes	.12(–.14-.39)	0.341	.11(–.17-.38)	0.4443
**IPV perpetration**	**OR(95%CI)**	**P-value**	**aOR(95%CI)**	**P-value**
Physical IPV				
ED	1.11(5.35–55.03)	0.216	1.13(.91–1.40)	0.285
Intervention	.30(.00–88.59)	0.676	.30(.00–100.52)	0.687
ED##Intervention	1.00(.88–1.14)	0.979	1.00(.87–1.15)	0.995
Gender attitudes	1.08(.96–1.23)	0.205	1.09(.94–1.26)	0.250
Emotional IPV				
ED	1.15(1.00–1.31)	0.047*	1.14(1.01–1.30)	0.036*
Intervention	196.82(.47– 81 609.29)	0.086	187.70(.55–63987.61)	0.079
ED##Intervention	.90(.77–1.04)	0.153	.90(.78–1.04)	0.154
Gender attitudes	1.20(1.04–1.38)	0.011*	1.25(1.06–1.48)	0.009*
Sexual IPV				
ED	1.13(.94–1.36)	0.195	1.15(.6–1.38)	0.141
Intervention	218.84(.07–680157.60)	0.189	276.64(.22–352016.10)	0.123
ED##Intervention	.87(.72–1.06)	0.166	.86(.72–1.03)	0.094
Gender attitudes	1.10(.94–1.28)	0.251	1.17(.92–1.48)	0.192
Economic IPV				
ED	1.05(.95–1.16)	0.371	1.04(.94–1.16)	0.412
Intervention	1.67(.02–113.66)	0.811	1.10(.01–92.93)	0.966
ED##Intervention	.98(.89–1.09)	0.761	.99(.88–1.11)	0.868
Gender attitudes	1.13(1.02–1.24)	0.015*	1.15(.99–1.34)	0.063
	**0(95%CI)**	**P-value**	**ap(95%CI)**	**P-value**
Combined IPV¶,§				
ED	.44(.18–.70)	0.002*	.37(.11–.64)	0.008*
Intervention	17.43(3.63–31.24)	0.015*	12.59(–.07–25.24)	0.051
ED##Intervention	–.44(–.77,–.12)	0.009*	–.33(–.63,–.03)	0.031*
Gender attitudes	.08(–.36–.53)	0.704	.23(–.11–.58)	0.177

* statistically significant *p* < 0.05, ED = emotional dysregulation, OR = Odds ratio, aOR = adjusted odds ratio, β = coefficient, aβ = adjusted coefficient, CI = confidence interval, ##=interaction. Difference in ED over time, gender attitudes and baseline outcomes were covariates in both models while adjusted models were further adjusted for age, education status and relationship status.

¶ denotes joint significance of the main effects (i.e. ED and intervention status) and their interaction term based Wald test (*p* < 0.05) for the adjusted models.

§ denotes significance of interaction term based Wald test (*p* < 0.05) for the adjusted models for the evidence of interaction.

## Data Availability

Data used to support this study are available from the corresponding author on reasonable request.
